# Clinical Analysis of Congenital Duodenal Obstruction and the Role of Annular Pancreas

**DOI:** 10.3390/medicina61020171

**Published:** 2025-01-21

**Authors:** Ümit Taşdemir, Oya Demirci

**Affiliations:** Department of Obstetrics, Division of Perinatology, Zeynep Kamil Women and Children Diseases Training and Research Hospital, İstanbul 34668, Turkey; demircioya@gmail.com

**Keywords:** duodenal obstruction, annular pancreas, ultrasound, prenatal diagnosis, chromosomal abnormality

## Abstract

*Background and Objectives*: Congenital duodenal obstruction (CDO) is a very rare anomaly with an incidence of 1 in 5000–10,000 live births. Annular pancreas is one of the reasons for CDO and is defined as the pancreatic tissue encircling the duodenum. The exact cause of annular pancreas remains unclear. *Materials and Methods*: A retrospective study was conducted on all prenatally diagnosed cases of CDO, with the diagnoses and ethiology confirmed by surgery after birth from 1 January 2018 to 1 January 2024. The cases suspected of having CDO in their fetuses underwent ultrasound evaluations on a weekly or biweekly basis. The cohort was divided into two groups, “CDO with annular pancreas” and “CDO without annular pancreas”, in order to compare the clinical characteristics and outcomes. *Results*: A total of 34 cases of CDO were detected prenatally, with 30 of these cases being confirmed through postnatal surgical interventions. The underlying ethiology was duodenal atresia in 15 cases (50%), duodenal web in 2 cases (6.6%) and annular pancreas in 13 cases (43.3%). All cases had a dilated stomach and double bubble sign. Polyhydramnios was identified in all cases except for one. Three cases were terminated and intrauterine demise was observed in one case. Nine of the cases (33%) died in the postnatal period. In 19 cases (55.9%), CDO was associated with chromosomal abnormalities. Chromosomal abnormalities were significantly more common in the cases of CDO with annular pancreas (*p* = 0.033). *Conclusions*: The prenatal diagnosis of CDO is mostly based on findings of double bubble and polyhydramnios. An annular pancreas, although rare, is an important cause of neonatal duodenal obstruction. An accurate diagnosis is usually performed during a laparotomy. Given the higher rates of chromosomal abnormalities in cases of annular pancreas, it is clear that more reliable markers or imaging techniques are needed to detect the ethiology of CDO in the prenatal period.

## 1. Introduction

Congenital duodenal obtruction (CDO) is a rare anomaly observed in the postnatal period with an incidence of 1 in 5000–10,000 live births [1]. Annular pancreas as a reason for CDO is defined as the presence of a partial or complete ring of pancreatic tissue encircling the duodenum, which is caused by the abnormal positioning of the embriyonic pancreas [2,3]. The incidence of annular pancreas is approximately 1 in 20,000 live births [4]. In pediatric populations, this condition is frequently associated with various congenital anomalies, such as Down syndrome, cardiac defects, duodenal atresia or stenosis, and intestinal malrotation [5]. Annular pancreatic tissue typically encircles the descending duodenum in proximity to the papilla of Vater; however, it is possible for other regions of the duodenum to be involved as well [6]. In approximately 25% of cases, the duodenum is completely encircled, while in around 75% of cases, it is only partially encircled [7]. Symptoms related to annular pancreas may differ significantly from one case to another. In some cases, it can result in severe symptoms soon after birth, while in others, it may remain asymptomatic for the entirety of a person’s life [8]. The severity of symptoms is associated with the level of compression that the annular pancreas applies to the duodenum.

An annular pancreas is responsible for approximately 30% of CDOs detected in neonates [9]. But it is crucial to emphasize that more than 40 percent of cases of annular pancreas lead to considerable duodenal obstruction and related complications [8]. As a result, when detected during prenatal evaluations, it acts as an important indicator of possible gastrointestinal emergencies. The ultrasound assessment of either partial or complete duodenal obstruction primarily relies on the identification of the double bubble sign, which is characterized by the concurrent distension of both the stomach and the duodenum. The existing literature indicates that a prenatal diagnosis of annular pancreas is typically performed in cases where there is significant duodenal obstruction, particularly when a double bubble sign is observable. It is important to recognize that numerous abdominal cystic masses (e.g., enteric duplication cysts) may also produce a double bubble appearance. The exact cause of annular pancreas remains unclear; however, recent research identified genetic factors associated with the HNF1B and FOXF1 genes [10,11].

The aim of the current study was to reveal the clinical characteristics and outcomes of the CDO cases and comparison of outcomes based on the ethiology.

## 2. Materials and Methods

### 2.1. Patient Selection

In this retrospective study, we evaluated all cases diagnosed with CDO prenatally, in which the diagnosis and ethiology were confirmed through surgical procedures in the postnatal period between 1 January 2018 and 1 January 2024. Inclusion criteria: (1) having a prenatal diagnosis of CDO and (2) having postnatally confirmed CDO and underlying ethiology by surgery or autopsy. Exclusion criteria: (1) having a confirmed CDO but no prenatal diagnosis and (2) having a prenatal diagnosis of CDO but no confirmation by surgery or autopsy.

### 2.2. Surveillance and Definitions

The cases suspected of having CDO in their fetuses underwent ultrasound evaluations on a weekly or biweekly basis to assess various critical parameters. These evaluations encompassed the amniotic fluid index; signs of fetal growth restriction; the presence of hyperechogenic bowel; assessments of the stomach, gallbladder, small intestines, colon and anal target sign; and the identification of pseudocysts or intra-abdominal calcifications. Furthermore, assessments were carried out to evaluate for fetal anemia, the presence of ascites and any associated abnormalities. The volume of amniotic fluid was evaluated through the amniotic fluid index, with measurements <50 mm categorized as oligohydramnios and those >250 mm categorized as polyhydramnios. Fetal growth restriction (FGR) was considered when the abdominal circumference measurement was <3rd centile. Intestinal structures that demonstrated isoechoic characteristics in comparison with neighboring bony elements were categorized as a hyperechogenic bowel. The diagnosis of fetal anemia was established when the peak systolic velocity of the middle cerebral artery exceeded 1.5 multiples of the median (MoMs).

This study did not establish a maximum gestational week limit regarding the timing of delivery. It was expected that cases would progress to spontaneous labor as long as the fetal well-being was maintained. In cases with fetal distress and in cases with a previous uterine scar, cesarean section was performed for all; otherwise, the mode of delivery was decided by the clinicians based on the labor’s progression.

### 2.3. Genetic Assessment

All patients received genetic counseling and were recommended to undergo prenatal genetic diagnostic testing for a fetal karyotype and chromosomal microarray (CMA) analysis (via Illumına platform). For those who chose not to have genetic diagnostic testing performed during pregnancy, these tests were carried out in the postnatal period. The CMA analysis was performed when the results of the karyotype analysis were normal; no further testing was carried out when karyotype anomalies were detected.

### 2.4. Confirmation of CDO and Assignments of Groups

The ethiology of CDO was confirmed in each case during surgical procedures performed postnatally. Intraoperative findings were documented through the surgical records. The cohort was divided into two groups in order to compare the outcomes based on the ethiology: CDO with annular pancreas and CDO without annular pancreas (duodenal atresia + duodenal web), in order to compare the clinical characteristics and outcomes.

The most recent status regarding the cases was acquired from examinations by pediatrics and pediatric gastroenterology specialists, as well as direct conversations with the parents.

All examinations were performed by experienced maternal–fetal medicine specialists utilizing a 5 MHz C4-8-D model convex abdominal transducer (VOLUSON E6, 2018 BT18, GE Healtcare, Zipf, Austria).

Descriptive data are presented as the median/range, mean ± standard deviation, numbers and percentages. Statistical analyses were performed using the Mann–Whitney U test for continuous data and the Fisher’s exact test for proportions. In all statistical analyses, the significance level (*p*-value) was determined at 0.05. The study data were analyzed using IBM SPSS statistics version 22.0 (IBM Corporation, Armonk, NY, USA). This study was approved by the local ethics committee (9 October 2024, decision no: 68).

## 3. Results

From 2018 to 2024, our perinatology center monitored a total of 34 cases of CDO, with 30 of these cases being confirmed through postnatal surgical interventions. The perinatal outcomes and ethiology of CDO of the cases are summarized in Table 1. Three (8.8%) cases were terminated at the request of the parents. One of these cases had isolated double bubble associated with trisomy 21; one had double bubble and nasal hypoplasia associated with trisomy 21; and the other had double bubble, double outlet right ventricle, unilateral renal agenesia and clenched hand associated with triploidy. Another case, which had double bubble, agenesis of the septum pellucidum and bilateral radial agenesis, along with a normal karyotype, resulted in intrauterine demise at 37 weeks of gestation. In these four (11.8%) cases, the CDO and its ethiology could not be confirmed because the families did not request a fetal autopsy.

Thirty cases of CDO, in which both the diagnosis and ethiology were established, were categorized into two distinct groups based on their ethiology: CDO with annular pancreas (13 cases) and CDO without annular pancreas (17 cases). The demographic and clinical characteristics, perinatal outcomes and their comparison between groups are summarized in Table 2. Accordingly, there were no significant differences between the two groups regarding age, parity, consanguineous marriage and gestational age at the time of diagnosis. Although the female gender was more common in the annular pancreas group, there was no significant difference between the groups in terms of gender (*p* = 0.259).

A prenatal genetic analysis was performed in only two cases. Karyotype and CMA analyses were conducted during the postnatal period for the other cases. The number of cases with chromosomal abnormality in both groups and their genetic results are summarized in Table 2. Accordingly, numerical and structural chromosomal abnormalities were detected in 10 cases (77%) with annular pancreas and in 6 cases (35.3%) without annular pancreas. A significant difference in terms of the chromosomal abnormality was noted between the two groups (*p* = 0.033).

The prenatal findings of ultrasonographic evaluations are summarized in Table 2. Fetal growth restriction was identified in six cases (46.2%) with annular pancreas and five cases (29.4%) without annular pancreas, with no significant difference between the groups. Polyhydramnios was noted in nearly all cases within the cohort, with the exception of a single case in the annular pancreas group (*p* = 0.433). Each case presented with a dilated stomach and the characteristic double bubble sign. We diagnosed CDO in all cases with the aid of the double bubble sign and did not observe any other noticeable findings to determine the underlying causes. Notably, none of the cases had hyperechogenic bowel; ascites; anemia; or any signs of intestinal perforation, including pseudocysts or intra-abdominal calcifications. Although associated anomalies were more common in the annular pancreas group, there was no significant difference between the two groups (*p* = 0.204). The gestational age at delivery, birthweight, hospital stay after surgery and rates of exitus in the long term were similar between groups (Table 2). A chromosomal abnormality was identified in six out of nine cases [four cases of trisomy 21, one case of 46,XX,(der 21) and one case of 47,XX,inv(9)(p11q13),+21] that resulted in death during the postnatal period. Among the remaining three cases, isolated CDO was found in two cases, as one case presented an umbilical systemic shunt. Death occurred the earliest on postnatal day 1 and the latest at 2.5 years of age.

## 4. Discussion

**Figure 1:**
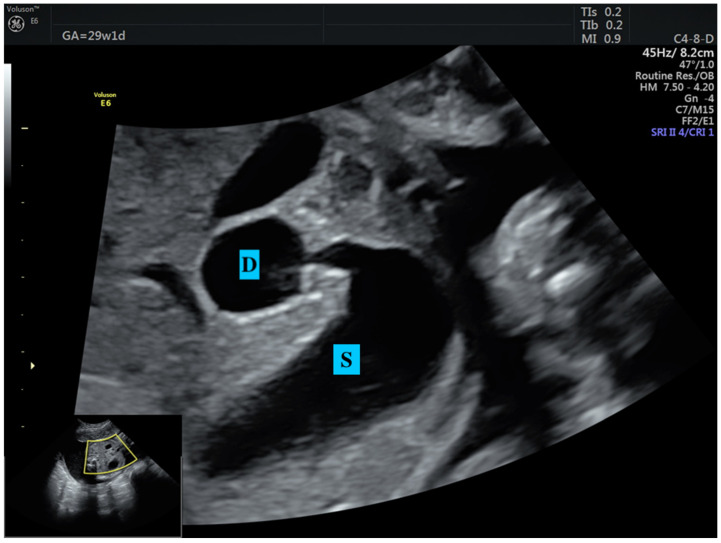
Double bubble sign at 30 weeks of gestation. The connection between the stomach and duodenum must be demonstrated to confirm the diagnosis. S: Stomach, D: Duodenum.

**Figure 2:**
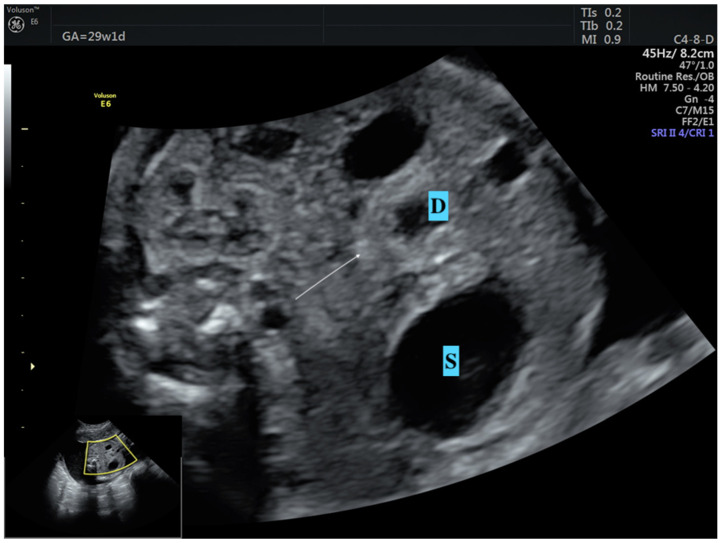
Prenatal sonographic appearance of annular pancreas at 30 weeks of gestation. Hyperechogenic band (arrow) surrounding duodenum (pliers sign) is the major finding of annular pancreas. S: Stomach, D: Duodenum.

Diagnosing CDO is usually uncomplicated, aided by unique ultrasonographic characteristics and key signs, like the double bubble sign (Figure 1). A similar double bubble appearance seen during an ultrasound examination can result from a range of pathological conditions, such as intestinal duplications and several other factors [12]. At this point, it is crucial to confirm that the cystic formation, thought to be the duodenum, is actually connected to the stomach (Figure 1). Therefore, identifying the underlying cause of the duodenal obstruction prior to birth is extremely important. This differentiation is essential for assessing the prognosis and for planning suitable interventions for the postnatal period. Nevertheless, determining the underlying cause through imaging methods continues to be a challenging endeavor, even after birth [13,14]. A study reported 100% accuracy in diagnosing annular pancreas prenatally in the case of double bubble and hyperechogenic band surrounding the duodenum (Figure 2) [12]. Recently, Yin et al. reported 80% sensitivity and 97% specificity of pliers sign diagnosing annular pancreas in the prenatal period [15]. However, these findings have not been confirmed in another study [16]. Consequently, accurately identifying the differential diagnosis for the underlying causes of CDO in the uterus remains a considerable challenge and is usually performed by surgical interventions in the postnatal period.

Although older studies have reported less than 5%, annular pancreas is responsible for approximately 30% of CDO, as documented in the current literature [9,16]. We found a 43% ratio of annular pancreas, which is slightly higher than previous studies. There are studies reporting a male preference in cases of annular pancreas [13,17]. In our study group, the female:male ratio was 19:11 in total and 10:3 in the annular pancreas group, indicating a female preferance. However, Wang et al. reported a female:male ratio of approximately 1 in annular pancreas cases [18]. In studies that involved a larger sample size, the ratio approached 1, which may suggest that there is no gender preferance in this condition.

Many studies in the literature reported a low birth weight in newborns affected by annular pancreas [19]. A recent study demonstrated that neonates affected by annular pancreas had a lower birth weight than those not affected [18]. The authors stated that an unknown factor affects intrauterine growth, especially prior to 30 weeks of gestation, and impaired fetal insuline secretion may have a role on this condition. The slowdown in growth rate, especially after birth, supports this hypothesis. In our study, we did not compare the birthweights of non-affected cases, but in both groups, we found low birth weights and fetal growth restriction in 11 cases (36.6%). Although there was no significant difference between the two groups, the lower mean birth weight (2167 ± 145 g vs. 2466 ± 167 g) in the annular pancreas group (13% difference) may suggest an altered pancreatic function. However, it is also well known that intestinal obstructions alone may affect fetal growth. Jejunoileal atresia and volvulus have been reported to be associated with fetal growth restriction [20]. Consequently, both factors may affect fetal growth in these cases.

CDO is associated with both chromosomal and structural abnormalities. Additional anomalies worsen the prognosis for both prenatal and postnatal outcomes [21]. Structural anomalies were found in approximately 50% of CDO cases in the current literature [9]. In our study, additional structural anomalies were detected in six cases (20%), mostly in the annular pancreas group. Associated chromosomal abnormalities were reported in the literature over a wide range between 8 and 63.9% [9,18,21], mostly with trisomy 21. Also, microdeletion abnormalities in microarray analysis was reported to be related with CDO [22]. We found chromosomal abnormalities in 16 cases (53.3%) in the cohort, in line with the literature. One of these cases had a 7q11.23 deletion, while another had Williams–Beuren syndrome, which is the first reported case in the literature that demonstrated a relationship with annular pancreas. Huang et al. previously reported three cases of Williams–Beuren syndrome, where one of the cases had suspected duodenal atresia, but they did not find the chance to confirm it [23]. More interestingly in our study, CDO with annular pancreas was significantly more associated (77% vs. 35.3%) with a chromosomal abnormality. This highlights the importance of the ethiology in CDO.

Our study reviewed the clinical characteristics of CDO cases and found different outcomes based on the ethiology. In our study group, the ultrasound detection of a double bubble sign was the only finding that aided in diagnosing CDO. We did not find any additional reliable indicators to identify the cause of obstruction. On the other hand, identifying the ethiology in the antenatal period may be more helpful in prenatal counseling.

## 5. Conclusions

The prenatal diagnosis of CDO is mostly based on findings of double bubble and polyhydramnios. Annular pancreas, although rare, is an important cause of neonatal duodenal obstruction. The accurate diagnosis of the underlying ethiology of CDO is usually performed during a laparotomy. Given the higher rates of chromosomal abnormality in cases of annular pancreas, it is clear that more reliable markers or imaging techniques are needed to detect the ethiology of CDO for a comprehensive counseling in the prenatal period.

## Figures and Tables

**Table 1:** Outcomes of 34 cases with double bubble.

Prenatal Diagnosis	n (%)
Duodenal obstruction	34 (100)
Chromosomal abnormalities	19 (55.9)
Perinatal outcomes	
Termination	3 (8.8)
Intrauterine demise	1 (2.9)
Live birth	30 (88.2)
Ethiology of duodenal obstruction in live births	
Duodenal atresia	15 (50)
Duodenal web	2 (6.6)
Annular pancreas	13 (43.3)

**Table 2:** Comparison of characteristics and perinatal outcomes of groups.

	CDO with Annular Pancreas (n = 13)	CDO Without Annular Pancreas (n = 17)	*p*-Value
Maternal characteristics			
Age, years (median/range)	34 (22–42)	33 (23–42)	0.509
Parity (median/range)	3 (1–9)	3 (1–5)	0.837
Consanguineous marriage (n, %)	2 (15.4)	4 (23.5)	
GA at prenatal diagnosis, weeks (median/range)	29 (22–37)	28 (24–33)	0.432
Mode of delivery			0.407
Vaginal (n, %)	2 (15.4)	6 (35.3)	
Cesarean (n, %)	11 (84.6)	11 (64.7)	
GA at delivery, weeks (median/range)	35 (33–40)	37 (27–41)	0.213
Fetal–neonatal characteristics			
Gender			0.259
Male (n, %)	3 (23)	8 (47)	
Female (n, %)	10 (77)	9 (53)	
Genetic result			
Normal (n, %)	3 (23)	11 (64.7)	
Trisomy 21 (n, %)	7 (53.8)	6 (35.3)	
7q11.23 deletion (Williams–Beuren) (n, %)	1 (7.7)	-	
46,XX,(der 21) (n, %)	1 (7.7)	-	
47,XX,inv(9)(p11q13),+21 (n, %)	1 (7.7)	-	
**Chromosomal abnormality (n, %)**	**10 (77)**	**6 (35.3)**	**0.033**
Ultrasonographic features			
Double bubble sign (n, %)	13 (100)	17 (100)	N/A
Dilated stomach (n, %)	13 (100)	17 (100)	N/A
FGR (n, %)	6 (46.2)	5 (29.4)	0.454
Early onset (n, %)	4 (30.7)	3 (17.6)	
Late onset (n, %)	2 (15.4)	2 (11.8)	
Polyhydramnios (n, %)	12 (92.3)	17 (100)	0.433
Associated anomaly (n, %)	4 (30.8)	2 (11.8)	0.204
Cardiac anomaly (n, %)	3 (23.1)	1 (5.9)	
Umbilical–systemic shunt (n, %)	1 (7.7)	-	
Esophageal atresia (n, %)	-	1 (5.9)	
Birthweight, grams (mean ± SD)	2167 ± 145	2466 ± 167	0.205
Delivery to surgery, days (median/range)	2 (0–10)	3 (0–16)	0.363
Hospital stay after surgery, days (median/range)	19 (10–68)	23 (15–90)	0.152
Current status in long term			0.443
Death (n, %)	5 (38.5)	4 (23.5)	
Living (n, %)	8 (61.5)	13 (76.5)	

Abbreviations: FGR, fetal growth restriction; GA, gestational age; N/A, non applicable.

## Data Availability

The data of this study are available upon reasonable request.
